# Use of absorbable hemostat bolster for prevention of donor renal artery kinking in kidney transplant

**DOI:** 10.3389/fsurg.2022.1032946

**Published:** 2022-11-29

**Authors:** Simon Hawlina, Blaž Orožen, Miha Arnol, Juš Kšela

**Affiliations:** ^1^ Department of Urology, University Medical Centre Ljubljana, Ljubljana, Slovenia; ^2^ Department of Surgery, Faculty of Medicine, University of Ljubljana, Ljubljana, Slovenia; ^3^ Department of Cardiovascular Surgery, University Medical Centre Ljubljana, Ljubljana, Slovenia; ^4^ Department of Nephrology, University Medical Centre Ljubljana, Ljubljana, Slovenia; ^5^ Department of Internal Medicine, Faculty of Medicine, University of Ljubljana, Ljubljana, Slovenia

**Keywords:** hemostat bolster, kinking, kidney transplantation, Doppler ultrasound, graft dysfunction, warm ischemia time

## Abstract

Transplant renal artery stenosis due to mechanical kinking is a rare but significant complication in kidney transplantation that can lead to graft dysfunction due to graft hypoperfusion, delayed graft function, or even global kidney infarction. When detected during surgery, re-anastomosis is usually performed after re-clamping, which inevitably prolongs the warm ischemia time, and increases the possibility of primary graft non-function. In this report, we describe a novel, noninvasive surgical technique whereby the donor renal artery is padded with absorbable hemostatic material (i.e., Surgicel) bolster, placed below the middle third of the renal artery in recipients who were found to have mechanical kinking during the implantation procedure. The bolster technique was used in 12 kidney transplant recipients who were found to have kinking of the donor artery during the primary surgery. After pillowing the renal artery with absorbable hemostatic bolster, no residual kinking was observed intra-operatively, and good allograft perfusion was confirmed with no Doppler ultrasound evidence of renal artery stenosis confirmed at 1 week, 1 month, and 1 year after transplantation.

## Introduction

Donor renal artery stenosis is an increasingly recognized complication after kidney transplantation. Reported incidence rates vary from 1% to more than 23% in the most rigorously screened recipients in the early, mid, and late post-transplant periods (1–4). Several mechanical, immune-related, systemic, and toxic pathophysiologic mechanisms, such as trauma to the intima, immune-related intimal proliferation, fibromuscular dysplasia and calcineurin inhibitor toxicity, have been identified as the most common factors contributing to the development of anastomotic or post-anastomotic stenosis in kidney transplant recipients (5–8). Donor renal artery stenosis secondary to mechanical kinking (i.e., a vessel curvature that creats a configuration with an impression of the vessel lumen into the inner curvature of the kink) is an extremely rare but crucial cause of post-anastomotic stenosis, leading to graft hypoperfusion and graft dysfunction (2, 3, 8–10). Although mechanical kinking is most commonly detected post-operatively, in some recipients kinking of the donor renal artery is detected intra-operatively, after the vascular clamps have been removed and the donor kidney has been placed in its final position (2, 3). In these clinical scenarios, most surgeons opt for re-clamping and re-anastomosis of the donor renal and recipient iliac vessels, prolonging the warm ischemia time and increasing the likelihood of primary graft non-function (11–13).

Accurate intraoperative recognition of the donor renal artery mechanical kinking and assessment of its clinical significance is difficult since the well-established Doppler flow measurements performed during surgery can be highly misleading and the surgeons' basic clinical judgement is often of limited value presumably due to an overestimation of surgical results (14). Thus, in order to properly diagnose mechanical kinking of the donor artery intraoperatively, the surgical team must rely on objective clinical signs of the graft macro- and micro-perfusion and potentially on transit time flow (TTF) measurements−mainly resistance index [defined as RI = (maximum volumetric peak flow−minimum volumetric peak flow)/maximum volumetric flow] and pulsatility index [defined as PI = (maximum volumetric peak flow−minimum volumetric peak flow)/mean volumetric flow] using lately developed transit time ultrasound technology, if available. A hypo-perfused kidney will show dark, livid discoloration and reduced turgor. Palpation of segmental arteries distal to the kinking site may reveal a weak or absent pulse compared with a strong pulse from the donor main renal artery proximal to the kinking site and in the region of the anastomosis. Additionally, RI > 0.7 and PI > 5 (both collected during intraoperative TTF measurements) are indicative of a high-grade obstruction in the blood flow through kinked donor renal artery.

In this report, we present a novel surgical technique in which absorbable hemostatic bolster is placed under the donor renal artery to support it, straighten its course, and establish unobstructed blood flow without the need to re-clamp and re-anastomose the affected vessels. The new technique was introduced by an experienced renal transplant surgeon who developed the method in collaboration with a cardiovascular surgeon and presented it to other renal transplant surgeons after three successful kinking resolutions. In our experience, this method has proven to be a simple, reliable, durable, and reproducible surgical technique to prevent arterial kinking in kidney transplant recipients.

## Patients and methods

This study was approved by the Medical Ethics Committee of the Republic of Slovenia and was conducted in full compliance with the principles of the Declaration of Helsinki. Because of the retrospective nature of the analysis, written informed consent was not obtained.

In our university tertiary medical center, which also serves as the only organ transplant referral center in our country, 521 kidney transplants were performed between 2012 and 2021. During this period, we noted postoperatively three cases (0.6%) of mechanical kinking (not present intraoperatively) of the donor renal artery, based on renal angiogram findings and supported by unsuccessful balloon angioplasty attempts, which required surgical correction with repositioning of the graft for definitive repair. All three grafts were lost.

In all recipients, the right or left iliac fossa was chosen as the site of implantation at the surgeon's discretion, depending on the characteristics of the patient and the graft. The length of the allograft artery and vein should match as closely as possible, but we were not successful in all cases, mainly because of individual differences in general anatomy, specific vessel configuration, and distribution of peripheral atherosclerotic disease in recipients and donors. A standard end-to-side vascular anastomosis technique under systemic heparinization with 5–0 or 6–0 Prolene sutures was used to anastomose the donor vessels to the recipient's iliac vasculature, depending on the characteristics of the vessel wall.

**Figure 1:**
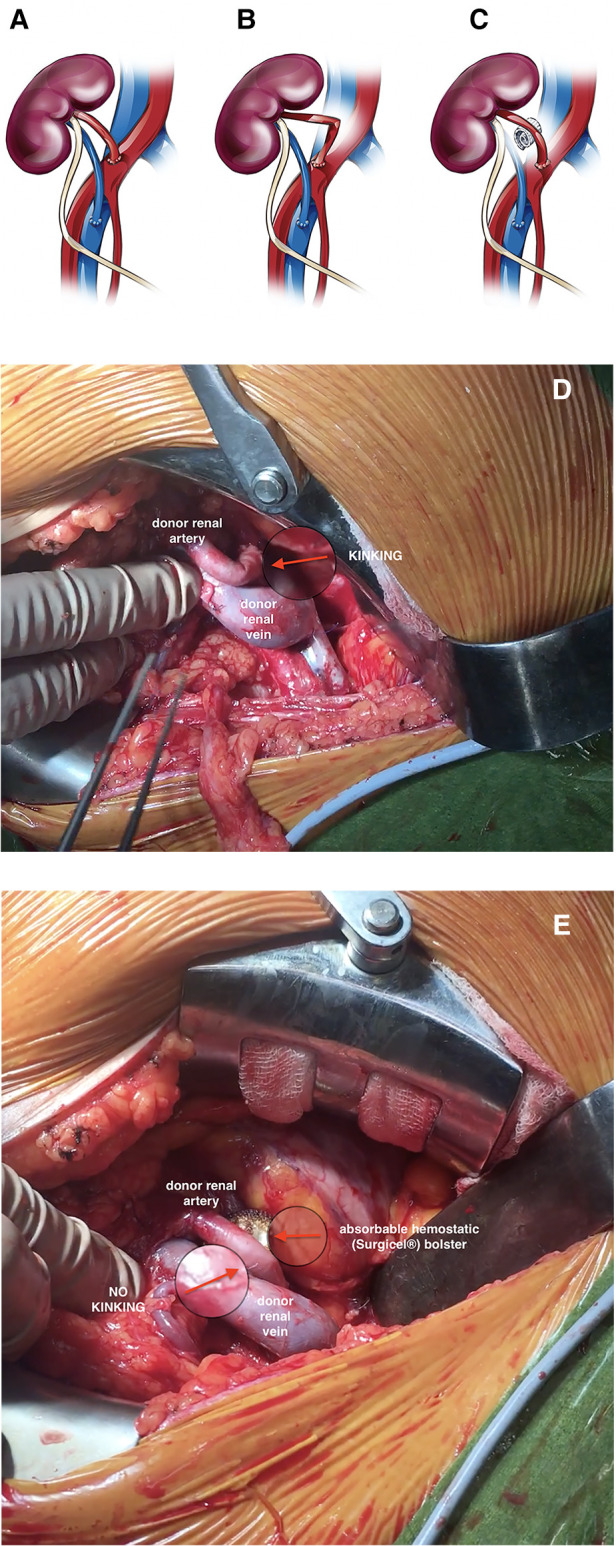
Schematic and real-life presentation of the use of an absorbable hemostatic pad to prevent kinking of the donor renal artery in kidney transplantation: normal anatomy (**A**), kinking of the donor renal artery (**B,D**), and an absorbable hemostatic bolster supporting the middle third of the kinked donor renal artery (**C,E**).

**Figure 2:**
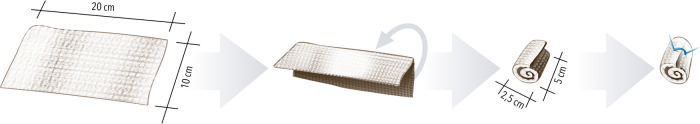
Schematic presentation of the bolster, made from surgicel that was manually folded, wrapped and rolled into compact bundle and latter secured with a suture to maintain the shape.

Mechanical kinking of the donor renal artery was detected intraoperatively in 12 recipients (events were evenly distributed among 15 transplant surgeons and occurred evenly over the observational period). To avoid arterial re-clamping and performing re-anastomosis, an absorbable hemostatic (Surgicel; Ethicon. Johnson and Johnson, Somerville, NJ, USA) bolster was placed under the middle third of the donor renal artery in all 12 cases to provide a pillow for the artery and straighten its course (Figure 1, Supplementary Video). The bolster was made by the surgeon from an original, commercially available fabric meshwork of absorbable hemostatic material that was manually wrapped and rolled lengthwise into compact bundle and later secured with a suture to maintain the shape of the bolster (Figure 2). When placing the bolster under the middle third of the donor renal artery, special care was taken not to generate unwanted compression of the donor renal or recipient iliac vein, which could lead to outflow obstruction and venous thrombosis of the transplanted kidney. Subsequently, kidney transplantation was performed in a standardized surgical manner, as previously reported elsewhere (15, 16). Briefly, after reperfusion of the kidney the allograft was placed into priorly dissected iliac fossa and ureteroneocystostomy performed by Lich-Gregoir technique using a 5/0 Monocryl absorbable suture. A 3 cm long submucosal tunnel was created as an anti-reflux procedure, and a 5 Fr 14 cm gauge double-J ureteral stent was routinely placed *in situ*. Lastly, following meticulous hemostasis a 15 Fr Blake drain was placed in the pararenal plain, and the surgical wound was closed in multiple layers (15, 16).

After the surgical procedure, all patients underwent routine Doppler ultrasound and conventional laboratory examinations at 1 week, 1 month, and 1 year after the primary surgery according to our kidney transplant center protocol to rule out potential early, intermediate, and long-term post-operative complications and allograft dysfunction. The resistance index, acceleration time, acceleration index, and serum creatinine levels were recorded at all regular follow-up examinations.

## Results

The values for the pre-selected Doppler ultrasound and laboratory parameters obtained at the 1-week, 1-month, and 1-year follow-up examinations are presented in Table 1. In all 12 patients, there were no Doppler signs of renal artery stenosis within the first year after transplantation, and the pre-selected parameters (resistance index, acceleration time, and acceleration index) were within the normal range at all follow-up examinations. Similarly, kidney graft function, as determined by serum creatinine levels, was appropriate in all 12 recipients at all follow-ups.

**Table 1:** Pre-selected Doppler ultrasound and laboratory parameters at 1 week, 1 month, and 1 year follow-up examinations after kidney transplantation.

	Post-transplant follow-up		
	1 week	1 month	1 year
Resistance index	0.75 ± 0.08	0.73 ± 0.04	0.75 ± 0.05
Acceleration time (ms)	42 ± 17	47 ± 19	32 ± 7
Acceleration index (m/s^2^)	6.27 ± 3.75	5.16 ± 2.70	10.79 ± 7.04
Serum creatinine (μmol/L)	268 ± 158	147 ± 43	174 ± 68

Data are presented as means ± standard deviation.

We did not observe any early, intermediate, or long-term vascular complications requiring surgical, endoscopic, or radiologic intervention (Clavien-Dindo Classification Grade III or IV complications), graft failure, or patient death (Clavien-Dindo Classification Grade V complication) in our recipient cohort.

## Discussion

Renal vascular complications are an important cause of morbidity and mortality in both living- and deceased-donor kidney transplantation. Among these complications, transplant renal artery stenosis is one of the leading causes of early graft dysfunction and failure. It usually occurs in 1% to 2.4% of patients after kidney transplantation (1), with an incidence of up to 23% in the most intensively monitored and studied recipients (1, 5, 8–10). Trauma to donor and recipient arteries during graft procurement and implantation resulting in intimal flaps, local dissections and increased intimal hyperplasia, inappropriate microsurgical techniques when creating the anastomosis, atheroma or advanced atherosclerosis, immunologically induced intimal proliferation, various systemic diseases such as fibromuscular dysplasia, and drug toxicities are the most commonly recognized factors contributing to the development of anastomotic or post-anastomotic stenosis in kidney transplant recipients (5).

In contrast to the causes already discussed, renal transplant artery stenosis due to mechanical kinking is a rare but significant complication that can lead to graft failure due to allograft hypoperfusion, delayed graft function, or even global kidney infarction (2, 3, 8). Mechanical renal artery kinking usually occurs when the length of the graft artery and the vein does not match, when the anastomotic side on the recipient iliac artery is not properly selected, and when the arterial graft is mispositioned when creating the anastomosis (5). It occurs more frequently in right kidney grafts, due to the natural discrepancy in vessel lengths; the renal vein is usually shorter than the renal artery (3). It has also been observed more frequently in end-to-end anastomoses, because of more vigorous dissection of the recipient's peri-vascular tissue, potentially higher tension at the anastomosis, and the need to connect smaller vascular lumens (1).

Although the specifics of the graft vascular anatomy, such as renal artery and vein length, optimal cuff alignment, and possible vascular anomalies, are routinely inspected at the back-table before graft implantation, the mechanical kinking of the donor artery becomes apparent only after the anastomosis is performed, the vascular clamps have been removed, and the donor kidney is placed in its final position. In such a clinical scenario, the renal vessels are usually re-clamped, and a new anastomosis is performed after the artery has been shortened, the cuff orientation has been optimized, or the anastomosis site has been re-positioned. However, all surgical procedures that include re-clamping and re-anastomosis of the renal vessels inevitably prolong the undesirable warm ischemia time once the transplanted kidney has already been perfused. This leads to additional ischemic insult to the transplanted organ, delayed graft function, or even primary graft non-function, and increases the risk of graft rejection (7, 11–13). Therefore, any surgical maneuver that avoids re-clamping of the renal vessels and minimizes warm ischemia time is welcome and strongly encouraged in the intra-operative resolution of arterial kinking. In this regard, the bolster technique described in our report may be a viable option in treating mechanical kinking without the need for re-clamping, thus allowing normalization and optimization of allograft perfusion without exposing it to undesirable warm ischemia. In our clinical practice, the bolster technique has proven to be a simple, reliable, durable, and reproducible surgical maneuver that can be used in a variety of anatomic settings in kidney transplantation.

Surgicel is an absorbable, oxidized cellulose material in a sterile fabric meshwork. When Surgicel is applied to the bleeding site, it swells into a brownish/black gelatinous mass that aids in the clotting process. As this agent lowers the pH of the surrounding tissue, lysis of red blood cells occurs, causing the dark discoloration. Although the mechanism of its action is not yet fully understood, it is mainly based on a mechanical compression effect. In addition, the polyanhydroglucuronic acid with a pH around 3 contained in the oxidized cellulose facilitates hemostasis by denaturing blood proteins and prevents bacterial growth (17, 18). Its biodegradation begins within 24 h, and depending on the amount used and the tissue bed, multinucleated giant cells appear within a week and are completely resorbed after 4–8 weeks, potentially leaving behind an inert fibrous-like tissue that can be found in unaltered form for several years to decades after the primary surgery (19–21). Although Surgicel is often left in surgical areas to provide effective postoperative hemostasis, previous reports have shown that swollen Surgicel can compress and interfere with the function of adjacent organs (22, 23), which was not observed in our patient cohort and is an important and meaningful finding. Indeed, all measured Doppler ultrasound parameters showed normal renal artery patency values up to one year after transplantation, indicating that the absorbable hemostatic material used to cushion the renal artery kept the artery in the correct position and did not lead to the formation of excessive fibrous tissue, that would lead to stenosis of the recipient's iliac artery or the donor's renal artery or impair blood flow to the graft. In addition, at our university tertiary medical center, all our patients are monitored regularly by ultrasound, even >1 year after transplantation, and the deterioration of graft function due to kinking would still be detected later. Although a commercially available fabric meshwork with the brand name Surgicel was used in all of our patients, we strongly believe that various absorbable hemostatic materials and/or products available on the market today or even retroperitoneal fat flap could be successfully used in this manner.

When cushioning the transplanted renal artery with an absorbable hemostatic cushion, special consideration must be given to venous outflow from the transplanted kidney. The bolster used must not interfere with the outflow through the renal and iliac veins and therefore must be placed in such a way that it does not cause stenosis of the venous system. If uncontrolled displacement of the hemostasis bolster is expected after the patient has been verticalized and moved normally, its primary position can be secured with a Prolene stitch during the surgical procedure. Venous stenosis could lead to venous thrombosis and graft failure, a devastating complication that significantly increases recipient morbidity and mortality after renal transplantation.

Although excellent clinical results were observed in all 12 reported patients, it is important to note that our report has some limitations. The first and most important limitation is that we did not have the opportunity to perform TTF measurements in the first 10 patients in whom the intraoperative diagnosis of arterial kinking was made solely on the basis of objective clinical signs of macro- and microperfusion of the graft (dark, livid discoloration of the poorly perfused allograft, decreased turgor, weak or absent pulse of segmental arteries with a strong pulse of the donor main renal artery proximal to the kinking side). Moreover, in these patients, resolution of mechanical kinking after pillowing the artery with absorbable hemostat was confirmed by clinical observation alone after achieving a light pink color of the transplanted organ with normal turgor and strong pulsations on the segmental arteries from the anastomosis to the renal hilum. In the last four years, intraoperative TTF measurements are routinely performed in all cases in which mechanical kinking is suspected, and PI > 5 is considered the cut-off value for the diagnosis of arterial kinking (14). Thus, in the last 8 patients from our report, TTF measurements were performed after arterial kinking was suspected based on clinical observation (Table 2). In all 8 cases, significant improvement of PI (12.9 ± 1.9 vs. 1.5 ± 0.4, *p* > 0.001, using Student's t-test) was observed after pillowing the donor artery with Surgicel bolster. TTF measurements showed decreased blood flow distal to the kink (PI of 12.9 ± 1.9) and improvement in the measured parameters after correction of the kinking with the hemostat-bolster technique described here (PI of 1.5 ± 0.4), which is now used at our institution in all renal recipients diagnosed intraoperatively with arterial kinking.

**Table 2:** Intraoperative pulsatility index measurements in last 8 patients in whom transit time flowmetry was performed before and after correction of the arterial kinking with the hemostat-bolster technique.

	Intraoperative transit time flow measurements (*n* = 8)		
	PI before	PI after	*p*
	Surgicel bolster	Surgicel bolster	
Patient 1	10.2	1.7	
Patient 2	13.7	2.1	
Patient 3	14.3	1.1	
Patient 4	11.8	1.5	
Patient 5	15.6	1.3	
Patient 6	10.5	2.0	
Patient 7	12.7	0.9	
Patient 8	14.1	1.6	
Mean ± SD	12.9 ± 1.9	1.5 ± 0.4	>0.001

IP, pulsatility index; SD, standard deviation.

## Conclusion

In our experience, cushioning the renal artery with an absorbable hemostatic bolster to straighten its course and provide support is a simple, reliable, durable, and reproducible surgical maneuver that does not affect the warm ischemia time. As seen in our patient cohort, the absorbable hemostatic material used to cushion the renal artery did not lead to stenosis of the recipient iliac artery or donor renal artery, which would compromise perfusion of the graft and provoke late renal artery stenosis. Therefore, we strongly believe that the hemostatic bolster technique is a viable surgical alternative to conventional renal artery re-clamping and re-anastomosis and can be used in a variety of anatomic situations in kidney transplantation.

## Data Availability

The raw data supporting the conclusions of this article will be made available by the authors, without undue reservation.
